# Separate 2-Incision Harvesting Technique for Bone–Patellar Tendon–Bone Autograft Using a Circular Hollow Burr in Arthroscopic Anterior Cruciate Ligament Reconstruction

**DOI:** 10.1016/j.eats.2025.103795

**Published:** 2025-08-26

**Authors:** Hyung-Suk Choi, Byung-Ill Lee, Kyoung-Dai Min, Jun-Bum Kim, Sai-Won Kwon, Yong-Beom Kim, Gi-Won Seo, Hyun-Uk Lee

**Affiliations:** a Department of Orthopaedic Surgery, Soonchunhyang University Seoul Hospital, Seoul, South Korea; b Department of Orthopaedic Surgery, Smarton Hospital, Bucheon, South Korea; c Department of Orthopaedic Surgery, Soonchunhyang University Bucheon Hospital, Bucheon, South Korea; d Department of Orthopaedic Surgery, Soonchunhyang University Cheonan Hospital, Cheonan, South Korea

## Abstract

Bone–patellar tendon–bone autografts are the gold standard for anterior cruciate ligament reconstruction but are associated with donor site morbidity. This Technical Note describes a separate vertical 2-incision approach using a circular hollow burr for bone–patellar tendon–bone autograft harvesting. The technique minimizes soft tissue and bone damage, reduces anterior knee pain and nerve injury, and improves cosmetic outcomes. The oscillating circular hollow burr enables precise bone plug extraction, enhancing graft integration and stability while protecting the articular cartilage. Our unique technique suggests a reliable alternative to traditional techniques, as well as provides a clinically effective and cosmetically favorable option, thus reducing donor site morbidity while minimizing risks associated with the harvesting procedure.

Graft selection remains a topic of debate in anterior cruciate ligament reconstruction. The bone–patellar tendon–bone (BPTB) autograft is historically regarded as the “gold standard” for anterior cruciate ligament (ACL) reconstruction due to its superior physiological and biomechanical advantages. These advantages include ease of harvesting, high stiffness, strength, strong graft integration, and lower laxity compared to other autografts.^1^, ^2^, ^3^ The BPTB autograft consistently provides excellent long-term stability and higher rates of return to sports in younger, active populations.^4^, ^5^, ^6^ However, despite these biomechanical advantages, traditional BPTB harvesting techniques are associated with donor site morbidity, including anterior knee pain, kneeling discomfort, and injury to the infrapatellar branch of the saphenous nerve, which can lead to numbness and reduced patient satisfaction, particularly in activities involving kneeling.^7^, ^8^, ^9^

To address these concerns, modifications to the traditional technique have been proposed, including a minimally invasive, 2-incision approach that preserves the mechanical advantages of the BPTB graft while reducing donor site morbidity.^3^^,^^10^^,^^11^ We describe the steps of a separated vertical 2-incision technique for BPTB autograft harvesting using an oscillating circular hollow burr system (Richard Wolf).

## Surgical Technique

### Indications and Contraindications

Indications for ACL reconstruction using the BPTB autograft include clinical and radiographic evidence of a complete ACL tear, particularly in younger, active patients engaged in pivoting sports that demand strong graft fixation and durability.^4^, ^5^, ^6^ The BPTB graft is particularly useful in patients requiring reliable knee stability and in cases where rapid graft integration and minimal laxity are prioritized.^4^^,^^5^^,^^12^

Contraindications for BPTB autograft harvesting include pre-existing patellar tendon pathology, such as patellar tendinopathy or previous fractures, which could compromise the structural integrity of the tendon and graft.^7^ Additionally, patients with severe patellofemoral arthritis or frequent anterior knee pain, or those involved in kneeling-intensive activities, may experience aggravated symptoms postoperatively, limiting the effectiveness of the BPTB graft.^7^^,^^8^^,^^13^

### Patient Positioning and Anesthesia

The patient is placed in the supine position with a lateral post, and the knee is flexed at 90° using a leg holder. This setup stabilizes the leg during graft harvesting and ACL reconstruction, providing optimal access to the patellar tendon and tibial tuberosity. A 90° flexion exposes the knee joint adequately while minimizing strain on the surrounding tissues. General anesthesia is administered with a femoral nerve block or adductor canal block to manage postoperative pain. Regional blocks significantly reduce anterior knee pain following surgery, enhance patient comfort, and decrease the reliance on opioids, thus reducing side effects such as nausea, vomiting, and sedation, further contributing to faster recovery and higher patient satisfaction.^14^, ^15^, ^16^

### BPTB Autograft Harvest Technique

After prepping and draping the knee, the separate vertical 2-incision technique using an oscillating circular hollow burr (Richard Wolf) is performed. The surgical sequence begins with the harvesting of the circular tibial bone plug, followed by the patellar tendon, and is completed with an oscillating circular patellar bone plug.

**Fig 1:**
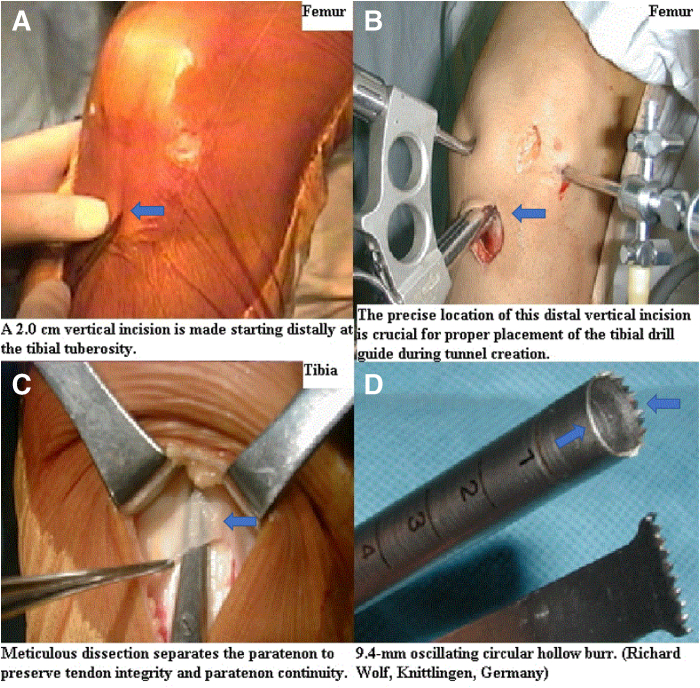
Initial steps of the separate vertical 2-incision technique for bone–patellar tendon–bone (BPTB) autograft harvesting and instrument characteristics. This figure illustrates the initial stages of the separate vertical 2-incision technique for harvesting BPTB autografts for arthroscopic anterior cruciate ligament (ACL) reconstruction, along with the features of a key instrument. (Patient position: supine, knee flexed; Surgical site: anterior aspect of the right knee.) (A) Distal vertical incision: Shows a 2.0-cm-long distal vertical incision extending proximally from the tibial tuberosity. This incision is initiated 1.0 cm medial to the tibial tuberosity and 2.0 cm distal to the joint line. (B) Tibial bone tunnel creation: Depicts the creation of the tibial bone through the distal vertical incision. The precise placement of this incision is crucial for proper positioning of the tibial drill guide during tunnel creation. (C) Paratenon preservation: Illustrates the careful evaluation and separation of the paratenon overlying the central third of the patellar tendon using Metzenbaum scissors through the distal vertical incision. This step is vital for preserving graft quality and promoting postoperative recovery. (D) Cutting edge of the 9.4-mm circular hollow burr: Compares the cutting edge of the 9.4-mm diameter circular hollow burr (Richard Wolf) used for graft harvesting (top) with a conventional instrument (bottom). This burr features sharp teeth on two-thirds of its circumference and a smooth surface on the remaining one-third, making it more effective in minimizing soft tissue damage and providing better protection to nerve structures compared to conventional instruments.

Starting distally at the tibial tuberosity and proceeding proximally, a 2.0-cm vertical incision is made, beginning 1.0 cm medial to the tibial tuberosity and 2.0 cm distal to the joint line (Fig 1A). The precise location of this distal vertical incision is crucial for proper placement of the tibial drill guide during tunnel creation (Fig 1B).

Lateral retraction of the distal vertical incision exposes the tendon attachment site. The central third of the patellar tendon attachment is measured, and meticulous dissection exposes the paratenon, which is incised and thoroughly separated from the patellar tendon to preserve the tendon’s structural integrity and maintain paratenon continuity (Fig 1C). A 10-mm-wide strip of the patellar tendon is harvested using a parallel double-bladed scalpel that cuts both sides of the patellar tendon simultaneously. Potential mismatches between the bone plugs and the tendon fibers should be considered, as malalignment can prevent all tendon fibers from being securely attached to both bone plugs, which can result in a weaker graft and compromise long-term outcomes.

**Fig 2:**
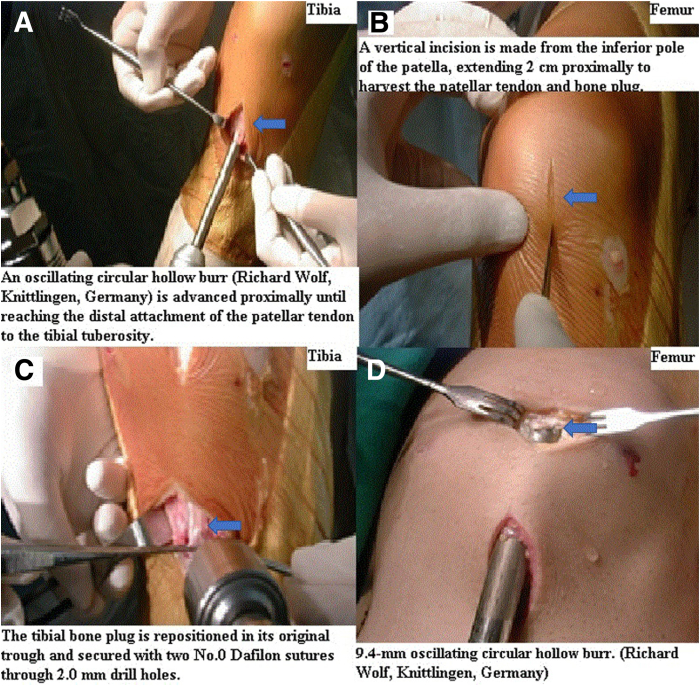
Tibial and patellar bone plug harvesting during bone–patellar tendon–bone (BPTB) autograft procurement. This figure illustrates the critical steps involved in harvesting both the tibial and patellar bone plugs during BPTB autograft procurement. (Patient position: supine, knee flexed; Surgical site: anterior aspect of the right knee.) (A) Tibial bone plug harvesting: Shows the harvesting of the tibial bone plug using a circular hollow burr attached to an oscillating compression air drill (Richard Wolf). This burr is advanced proximally until it reaches the distal tibial attachment of the patellar tendon. (B) Proximal vertical incision: Depicts a separate proximal vertical incision commencing at the inferior pole of the patella and extending 2.0 cm proximally along the midline of the patella. This incision facilitates the harvesting of the patellar tendon and patellar bone plug. (C) Repositioning and suture preparation of the tibial bone plug: Illustrates the repositioning of the harvested tibial bone plug into its original bony trough, followed by the creation of 2 drill holes (indicated by arrows) using a 2.0-mm drill, through which nonabsorbable polyamide monofilament surgical sutures (No. 0 Dafilon; B.Braun Surgical, S.A.) are passed. (D) Patellar bone plug harvesting: Shows the harvesting of the patellar bone plug by advancing the oscillating circular hollow burr from distal to proximal. This process is performed while minimizing damage to the articular surface of the patella.

To optimize tibial bone plug integration, a bone plug (20-25 mm in length, 8.0 mm in depth) is harvested through a distal vertical incision using a 9.4-mm diameter oscillating circular hollow burr (Richard Wolf) (Fig 1D) at an approximately 65° angle. An oscillating circular hollow burr is advanced proximally until it reaches the distal attachment of the patellar tendon to the tibial tuberosity (Fig 2A). Care is taken to avoid excessive bone and soft tissue damage to minimize the risk of complications, such as tibial fractures. The circular tibial bone plug is repositioned in its original bony trough, two 2.0-mm drill holes are made, and 2 nonabsorbable polyamide monofilament surgical sutures, No. 0 Dafilon (B.Braun Surgical, S.A.), are passed through, with no additional manipulation of the bone plug required (Fig 2B).

A separate proximal vertical incision begins at the inferior pole of the patella and extends 2.0 cm proximally over the midline of the patella, facilitating the harvesting of the patellar tendon and patellar bone plug (Fig 2C).

Before harvesting the patellar bone plug, the correct orientation of the BPTB graft must be verified to prevent twisting or malalignment. Special attention should be given to the integrity of the paratenon and patellar tendon in the concealed zone between 2 incisions. This assessment, performed with Metzenbaum scissors, ensures the paratenon remains intact and is properly separated from the patellar tendon. This step is critical for preserving graft quality and promoting better conditions for postoperative healing.

**Fig 3:**
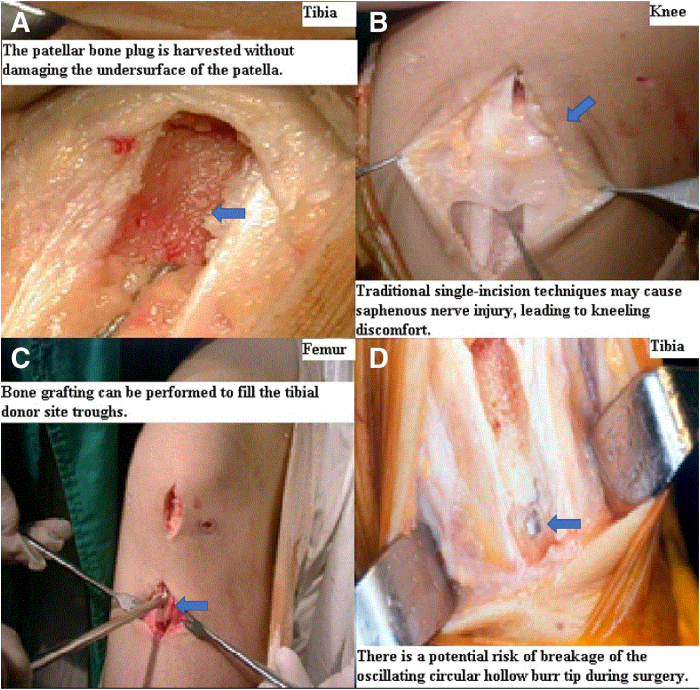
Outcomes, comparison, and potential complications of the bone–patellar tendon–bone (BPTB) autograft harvesting technique. This figure presents the outcomes following BPTB autograft harvesting, a comparison with traditional techniques, and potential complications. (Patient position: supine, knee flexed; Surgical site: anterior aspect of the right knee.) (A) Preservation of patellar articular surface: Shows the intact preservation of the patellar articular undersurface after bone plug harvesting. This highlights a significant advantage of the described technique. (B) Traditional single-incision technique: Shows the harvesting of the BPTB tendon through a traditional longitudinal single incision. This method contrasts with the separate 2-incision technique presented in this article. (C) Autogenous cancellous bone for grafting: Illustrates that a sufficient amount of circular-shaped autogenous cancellous bone can be obtained during bone tunnel reaming. This cancellous bone can be utilized for bone grafting in areas of bone defect, offering an additional benefit. (D) Risk of circular hollow burr tip breakage: Depicts an instance where the tip of the circular hollow burr broke during surgery. This represents a rare but potential complication, emphasizing the importance of proper training and skilled surgical technique.

Once graft orientation and paratenon integrity are confirmed, the free end of the two 2.0-mm drill holes are made, and 2 nonabsorbable polyamide monofilament surgical sutures, No. 0 Dafilon (B.Braun Surgical, S.A.), passing through the tibial bone plug are gently pulled in a retrograde direction through a 9.4-mm diameter oscillating circular hollow burr (Richard Wolf) with a 24-gauge flexible guidewire. Simultaneously, the oscillating circular hollow burr is advanced from distal to proximal to harvest the patellar bone plug (15.0-20.0 mm in length, 8.0 mm in depth), with proximal separation of the patellar bone plug achieved by elevating the direction of the oscillating circular hollow burr (Fig 2D). This careful step ensures that the patellar bone plug is harvested without damaging the undersurface of the patella (Fig 3A), resulting in a more consistent graft and reducing the risk of patellofemoral complications. Gentle distal direction force is applied to ensure smooth extraction. Without the need for instruments such as Cocker’s clamps or forceps, the patellar bone plug, patellar tendon, and tibial bone plug are removed through the distal vertical incision. The entire BPTB autograft, contained within the circular hollow burr lumen, is then fully retrieved, completing the harvesting procedure (Video).

## Discussion

The success of ACL reconstructions largely depends on several key factors: proper patient selection, thorough preoperative planning, selection of appropriate grafts tailored to individual patients, creation of anatomically precise bone tunnels on the footprints, secure graft fixation, and patient-specific postoperative rehabilitation. While no single graft option consistently shows a clear advantage over the others, BPTB autografts remain one of the most commonly used autograft sources. Despite their excellent outcomes, BPTB autografts often result in significant donor site morbidity.^17^ Patients frequently report more postoperative and long-term anterior knee pain compared to those who receive hamstring autografts.^14^ Zhou et al.^15^ reported a 34% incidence of dysesthesia and kneeling pain after ACL reconstruction with the BPTB autograft. Additionally, the BPTB graft harvest carries a 0.2% to 1.3% risk of patellar fracture.^16^^,^^18^ Anatomic studies indicate that variations in the saphenous nerve trajectory in the anterior knee region pose a risk of iatrogenic injury to its infrapatellar branch, resulting in focal hypoesthesia, dysesthesia, or the development of symptomatic painful neuromas.^19^, ^20^, ^21^

The 2-incision technique for BPTB autograft harvesting is not a new concept, having been previously introduced by earlier authors.^5^^,^^15^^,^^22^ Traditional single-incision techniques have been associated with higher rates of complications, including anterior knee pain and injury to the infrapatellar branch of the saphenous nerve, leading to discomfort during activities like kneeling^9^^,^^18^^,^^19^ and prolonged recovery times (Fig 3B). Its resurgence is driven by the effort to reduce donor site morbidity. Studies consistently show that the 2-incision technique results in fewer complications, including reduced anterior knee pain, less numbness, and improved functional outcomes, compared to the single-incision technique.^3^^,^^5^^,^^20^ Tsuda et al.^5^ recommend horizontal incisions combined with careful retinacular dissection to minimize nerve injury risk, while Kartus et al.^11^ and Gaudot et al.^3^ advocate for vertical incisions, particularly for future revision surgery considerations. The 2-incision technique preserves soft tissue integrity and minimizes nerve injury, resulting in faster recovery and better functional outcomes. Akoto et al.^23^ reported the use of a specialized burr in quadriceps autograft harvesting, similar to our application of the oscillating circular hollow burr in BPTB autograft harvesting.^24^

Our approach builds on this technique by incorporating the use of an oscillating circular hollow burr system (Richard Wolf). The oscillating circular hollow burr provides advantages over traditional tools, such as osteotomes and oscillating saws, in terms of surgical efficiency and bone plug accuracy. The oscillating circular hollow burr enables smooth extraction of circular bone plugs, eliminating the need for extensive manual shaping, which is usually required with conventional instruments. This reduction in manual shaping not only streamlines the procedure but also decreases the risk of bone fractures or plug malalignment.^25^

Furthermore, the oscillating circular hollow burr (Richard Wolf) reduces overall tissue trauma. When combined with the separate vertical 2-incision technique, it protects the paratenon and preserves tendon integrity. Another significant advantage is that the oscillating circular hollow burr (Richard Wolf) has a protective effect on the articular undersurface of the harvested area. Traditional instruments may increase the risk of damage, potentially leading to long-term complications such as patellofemoral arthritis. The smoother, more precise cuts provided by the oscillating circular hollow burr help reduce the risk of degenerative changes, which is particularly important for active patients.^26^^,^^27^

Moreover, the separate vertical 2-incision technique, combined with the reduced soft tissue dissection facilitated by the oscillating circular hollow burr, increases the likelihood of nerve preservation. This approach lowers the risk of postoperative numbness and anterior knee pain,^28^ and it is particularly important for patient comfort and functional recovery.

Finally, the oscillating circular hollow burr (Richard Wolf) improves graft integration. The circular bone plug provides more optimal bone contact and secure fit within the tibial and femoral tunnels, which is crucial for patients with high physical demands, such as athletes, as it ensures stronger graft fixation and reduces the risk of graft failure. Additionally, the smooth circular bone plug reduces stress concentration, particularly in the patellofemoral joint, which enhances long-term outcomes and minimizes the risk of patellofemoral arthritic changes (Table 1).^29^

**Table 1:** Advantages and Disadvantages of the Separate Vertical 2-Incision Harvesting Technique for Bone–Patellar Tendon–Bone Autograft Using a Circular Hollow Burr

**Advantages** Reduced donor site morbidity (e.g., less anterior knee pain, kneeling discomfort) Enhanced nerve protection, lowering the risk of postoperative numbness More precise bone plug harvesting, minimizing the risk of fractures or malalignment Improved graft integration particularly important for active patients Potential to use harvested cancellous bone for donor site grafting **Disadvantages** Limited visually due to small incisions Requires specialized instruments, potentially limiting availability Risk of burr tip breakage during surgery Technically challenging, particularly during the early practical phase

Interestingly, after harvesting, adequate quantities of cylindrical cancellous bone can be obtained as a single unit to address both patellar and tibial donor site bone defects through reaming tunnels. Consequently, bone grafting can be performed to fill the patellar and tibial donor site troughs (Fig 3C).

Despite several clear advantages, our technique is not without its drawbacks, including limited visibility during the procedure. The separate vertical 2-incision technique requires careful planning and precise execution, as technical challenges may arise, particularly during the early stages of practical application, due to the small incisions and limited dissection. Additionally, the need for special instruments, such as an oscillating hollow burr (Richard Wolf), poses a risk of the burr tip breaking during surgery (Fig 3D).^23^ However, with proper training and surgical expertise, these challenges can be mitigated. Future studies focusing on complication rates, graft integration, and functional outcomes over time with longer follow-up periods are essential to fully assess the long-term practical benefits of this technique. This Technical Note describes a separate vertical 2-incision BPTB autograft harvesting technique utilizing a circular hollow burr, highlighting its clinical relevance and the potential benefits of enhanced patient satisfaction, improved clinical outcomes, and minimized donor site morbidity (Table 2).

**Table 2:** Pearls and Pitfalls

**Pearls** The separate vertical 2-incision technique reduces donor site morbidity by preserving soft tissue and minimizing nerve injury, especially around the saphenous Preserving paratenon during patellar tendon dissection ensures graft quality and supports better postoperative healing Precise use of the oscillating circular hollow burr, accurate positioning, and careful angulation during bone plug harvesting and extraction are critical for optimal graft integration, allowing for smooth extraction of bone plugs, minimizing shaping, and reducing the risk of fractures or malalignment Bone grafting using circular cancellous bone harvested from reamed tunnels, resulting in better postoperative healing **Pitfalls** Nerve injury risks particularly to the infrapatellar branch of the saphenous nerve, if dissection is not meticulous, particularly in the concealed zone, leading to postoperative numbness or dysesthesia Excessive force during bone plug extraction increases the risk of tibial or patellar bone plug fractures or damage to the undersurface of the patella Instrument failure, such as burr tip breakage, poses a risk if not used with caution, particularly in dense bone or with improper angulation

## Disclosures

All authors (H-S.C., B-I.L., K-D.M., J-B.K., S-W.K., Y-B.K., G-W.S., H-U.L.) declare that they have no known competing financial interests or personal relationships that could have appeared to influence the work reported in this paper.

## Supplementary Data

Video 1 This video shows a separate vertical 2-incision technique for harvesting bone–patellar tendon–bone autografts during arthroscopic anterior cruciate ligament reconstruction. It specifically showcases the entire process of graft harvesting utilizing a circular hollow burr (Richard Wolf). The video commences with the initial incision near the distal tibial tuberosity and proceeds through the second incision at the inferior pole of the patella. This technique offers distinct advantages over traditional single-incision methods, including minimized donor site morbidity, reduced risk of nerve injury, and more precise bone plug harvesting. Each surgical step is presented with clear visual guidance, providing essential educational insights for mastering the technique.
